# Supplementary material for: Herbaria macroalgae as a proxy for historical upwelling trends in Central California
Source: Proc Biol Sci. 2020 Jun 17;287(1929):20200732. doi: 10.1098/rspb.2020.0732 (PMC7329038; doi:10.1098/rspb.2020.0732)
Supplement: Supplementary material for “Herbaria macroalgae as a proxy for historical upwelling trends in Central California” [file rspb20200732supp1.pdf]

Supplementary material for “Herbaria macroalgae as a proxy for historical upwelling trends in Central California”

Emily A. Miller[1]*, Susan E. Lisin[1], Celia M. Smith[2], Kyle S. Van Houtan[1,3]

[1] Monterey Bay Aquarium, 886 Cannery Row, Monterey, CA 93950, USA; [2] University of Hawai'i at Manoa, 3190 Maile Way, Honolulu, Hawai'i 96822, USA; [3] Nicholas School of the Environment, Duke University, Box 90328, Durham, NC 27708, USA.

DOI: 10.1098/rspb.2020.0732

Supplementary Methods

Eight archived specimens, from the first half of the time series, listed only the city as a location. Based on the distribution of collecting in that time, they were likely from our focal area. Collectors of the two oldest specimens recorded the year but not date. They were excluded from analyses examining seasonal effects but included in all other analyses and were assigned the middle date in the year July 1st which is the common period for seaweed collecting (54% of our *Gelidium* specimens were collected May – July). Four specimens listed a June – July collection period and were included in all analyses, using July 1st for these dates.

We homogenized macroalgal tissues and shipped them to three labs. Amino acid analyses required 0.05 g, heavy metals 0.5 g, and stable isotope analyses 0.05 g.

Texas A&M University Protein Chemistry Laboratory (TAMU PCL) performed amino acid analyses using acid liquid-phase hydrolysis (6N HCl in a PicoTag Workstation) followed by exposure to 100 °C for 22 hours. After hydrolysis, TAMU PCL filtered and dried samples, removed HCl, and reconstituted them in 0.4 M borate buffer. An Agilent 1260 HPLC then derivatized and separated amino acids with o-phthalaldehyde and 9-fluoromethyl-chloroformate. A G1356D variable wavelength detector or a G1321B in-line fluorescence detector then detected amino acids by UV absorbance or fluorescence, respectively.

Amino acid and total protein values were reported in % by weight. TAMU PCL quantified the % by weight composition for the amino acids: alanine (ALA), arginine (ARG), aspartic acid/asparagine (ASX), glutamic acid/glutamine (GLX), glycine (GLY), histidine (HIS), isoleucine (ILE), leucine (LEU), lysine (LYS), methionine (MET), phenylalanine (PHE), proline (PRO), serine (SER), threonine (THR), tyrosine (TYR), and valine (VAL).

California Animal Health & Food Safety Laboratory System (CAHFS) at UC Davis performed heavy metal screens by digesting algal tissue with 3 mL of nitric acid at 190° C. After digestion, CAHFS added 2 mL of hydrochloric acid and brought the sample to 10 mL with 18 Mohm water. They analyzed the solution by inductively coupled plasma optical emission spectrometry. CAHFS digested and analyzed a method blank, laboratory control spike, sample over-spike, and a certified reference material from the National Research Council of Canada with each batch to ensure data quality. A drift check every ten samples ensured instrument stability. When a metal was undetected, we used the detection limit as the value.

Heavy metal concentrations were reported in parts per million (ppm). The heavy metal panel screened for lead (Pb), manganese (Mn), iron (Fe), mercury (Hg), arsenic (As), molybdenum (Mo), zinc (Zn), copper (Cu), and cadmium (Cd).

Texas A&M University Stable Isotopes for Biosphere Science Laboratory (TAMU SIBS) performed stable isotope analyses. TAMU SIBS combusted samples in a Costech Elemental Combustion System with a Thermo Scientific Delta V Advantage mass spectrometer in continuous flow mode for carbon and nitrogen isotope analyses. Calibration was performed using USGS glutamic acid-40 and -41. TAMU SIBS calculated %N and %C using National Institute of Standards and Techonology plant standard Apple1515. Two in-house plant standards, SIBS-pCo and SIBS-pEc provided quality control. A Thermo Scientific EA IsoLink coupled to a Conflo IV and a Thermo Scientific Delta V Advantage mass spectrometer analyzed oxygen isotope ratios using IAEA and USGS standards.

TAMU SIBS analyzed samples in duplicate. All carbon, nitrogen, and oxygen isotope data are reported in δ notation in per mil (‰). $\delta X = R_{sample} = R_{standard} - 1$; where $X = {}^{13}C$, ${}^{15}N$, ${}^{18}O$ and $R = {}^{15}N/{}^{14}N$ or ${}^{13}C/{}^{12}C$ or ${}^{18}O/{}^{16}O$. The standards used were Vienna Pee Dee Bolemnite (VPDB) for $\delta^{13}C$ [1], international atmospheric N_2 for $\delta^{15}N$ [2], and IAEA-CH-3 cellulose for $\delta^{18}O$ [3].

1. Coplen T.B., Brand W.A., Gehre M., Groning M., Meijer H.A.J., Toman B., Verkoutere R.M. 2006 New guidelines for δ13C measurements. *Analytical Chemistry* **78**(7), 2439-2441.
2. Mariotti A. 1983 Atmospheric nitrogen is a reliable standard for natural 15N abundance measurements. *Nature* **303**, 685-687.
3. Saurer M., Robertson I., Seigwolf R., Leuenberger M. 1998 Oxygen isotope analysis of cellulose: an interlibrary comparison. *Analytical Chemistry* **70**, 2074-2080.

Supplementary Tables and Figures

Table S1. Curing experiment result summaries of stable isotopes over one year.
Table S2. Composition of archived and modern herbarium paper used in specimen curing experiment.
Figure S1. Map of study region including collecting site for curing experiment and collecting region for historical herbarium specimens.
Figure S2. Amino acid composition of macroalgae pressed on archived and modern paper over one year curing experiment.
Figure S3. Heavy metal concentration of macroalgae on archived (top) and modern (bottom) paper over one year curing experiment.
Figure S4. Total protein composition of macroalgae on archived and modern paper combined over one year curing experiment.
Figure S5. Stable isotope values of macroalgae on archived and modern paper combined by species over one year curing experiment.
Figure S6. $\delta^{15}N$ values of *G. purpurascens* collected in southern Monterey Bay and sardine catch in Monterey were not correlated over the 140 year period.

Figure S7. $\delta^{18}O$ values of *G. purpurascens* collected in southern Monterey Bay and sea surface temperature collected at Hopkins Marine Station were not correlated over the 140 year period. Loess models were fit to each data set to visualize local trends.
Figure S8. $\delta^{18}O$ values of *G. purpurascens* collected in southern Monterey Bay and calculated PDO index values for the region were not correlated over the 140 year period.
Figure S9. Modeled fits of stable carbon isotope ratios ($\delta^{13}C$) against modeled upwelling index values with varying spans.
Figure S10. Modeled fits of stable nitrogen isotope ratios ($\delta^{15}N$) against modeled upwelling index values with varying spans.
Figure S11. Modeled fits of stable oxygen isotope ratios ($\delta^{18}O$) against modeled upwelling index values with varying spans.
Figure S12. Optimal spans for loess models of historical macroalgae specimens and loess models of upwelling index values.
Figure S13. Relationship of stable isotope ratios from herbarium specimens to Bakun upwelling index.

Supplementary Datasets

Dataset S1. Marine macroalgae specimens collected for curing experiment.

Dataset S2. Amino acid, total protein, and heavy metal summaries from the curing experiment over one year. Amino acid and total protein values are reported in % by weight. Heavy metal values are concentrations (ppm). Values are averaged across paper types. Macroalgae species: *C. columbiana* (CC), *C. ruprechtiana* (CR), *G. purpurascens* (GP), *M. pyrifera* (MP), *S. compressa* (SC), and *U. californica* (UC). Amino acids: alanine (ALA), arginine (ARG), aspartic acid/asparagine (ASX), glutamic acid/glutamine (GLX), glycine (GLY), histidine (HIS), isoleucine (ILE), leucine (LEU), lysine (LYS), methionine (MET), phenylalanine (PHE), proline (PRO), serine (SER), threonine (THR), tyrosine (TYR), and valine (VAL).

Dataset S3. Historical herbaria specimens used in hindcasting case study.

Dataset S4. Macroalgae amino acid and total protein data from curing experiment.

Dataset S5. Macroalgae heavy metal data from curing experiment.

Dataset S6. Macroalgae stable isotope data from curing experiment.

Dataset S7. Macroalgae stable isotope data from herbarium specimens.

Table S1. Stable isotope summaries from the curing experiment over one year. Values incorporate macroalgae samples pressed on both archived and modern paper.

species	isotope ratio	mean (‰)	sd	min	max
C. columbiana	$\delta^{13}C$	-14	1	-15.9	-12.2
C. ruprechtiana	$\delta^{13}C$	-30	1	-32.3	-28.3
G. purpurascens	$\delta^{13}C$	-16	1.9	-20.1	-13.1
M. pyrifera	$\delta^{13}C$	-12.6	0.9	-14.2	-11.3
S. compressa	$\delta^{13}C$	-15.7	0.9	-17.4	-14.25
U. californica	$\delta^{13}C$	-13	0.5	-14.4	-11.7
C. columbiana	$\delta^{15}N$	8.7	0.6	7.8	10.5
C. ruprechtiana	$\delta^{15}N$	9	1	8.2	11.1
G. purpurascens	$\delta^{15}N$	7.2	0.9	5.5	8.6
M. pyrifera	$\delta^{15}N$	11.1	0.5	11.1	13
S. compressa	$\delta^{15}N$	9.3	0.8	8.2	11.1
U. californica	$\delta^{15}N$	8.6	0.3	8.1	9.5
C. columbiana	$\delta^{18}O$	20.5	2.2	16.6	24.4
C. ruprechtiana	$\delta^{18}O$	14.7	1.6	12.2	16.9
G. purpurascens	$\delta^{18}O$	22.3	1.4	19.3	23.7
M. pyrifera	$\delta^{18}O$	18.7	1	17	20.4
S. compressa	$d^{18}O$	19.5	1.5	14.7	20.1
U. californica	$\delta^{18}O$	17.9	1.5	14.7	20.1

Table S2. Composition of archived and modern herbarium paper used in specimen curing experiment. Means across replicates are reported.

variable	archived paper	modern paper	reporting limit (heavy metals)
stable isotope ratio (‰)			
$\delta^{13}C$	-23.8	-24.8	
$\delta^{15}N$	content too low to be determined		
$\delta^{18}O$	29.0	26.8	
total protein and amino acids (% by weight)			
total protein	1.1	0.0	
ASX	8.2	9.8	
GLX	14.8	21.3	
SER	4.5	0	
HIS	0.1	0	
GLY	31.6	9.1	
THR	2.0	5.0	
ALA	11.8	7.1	
ARG	9.6	10.9	
TYR	0	5.7	
VAL	3.6	10.4	
MET	0.1	0	
PHE	2.6	4.4	
ILE	2.1	3.9	
LEU	4.5	12.4	
LYS	4.4	0	
PRO	0	0	
heavy metals (ppm)			
Pb	2.8	nd	2
Mn	4	1.7	0.2
Fe	86	34	2
Hg	nd	nd	2
As	nd	nd	2
Mo	nd	nd	0.8
Zn	44	4.3	0.6
Cu	6.6	0.93	0.6
Cd	nd	nd	0.6

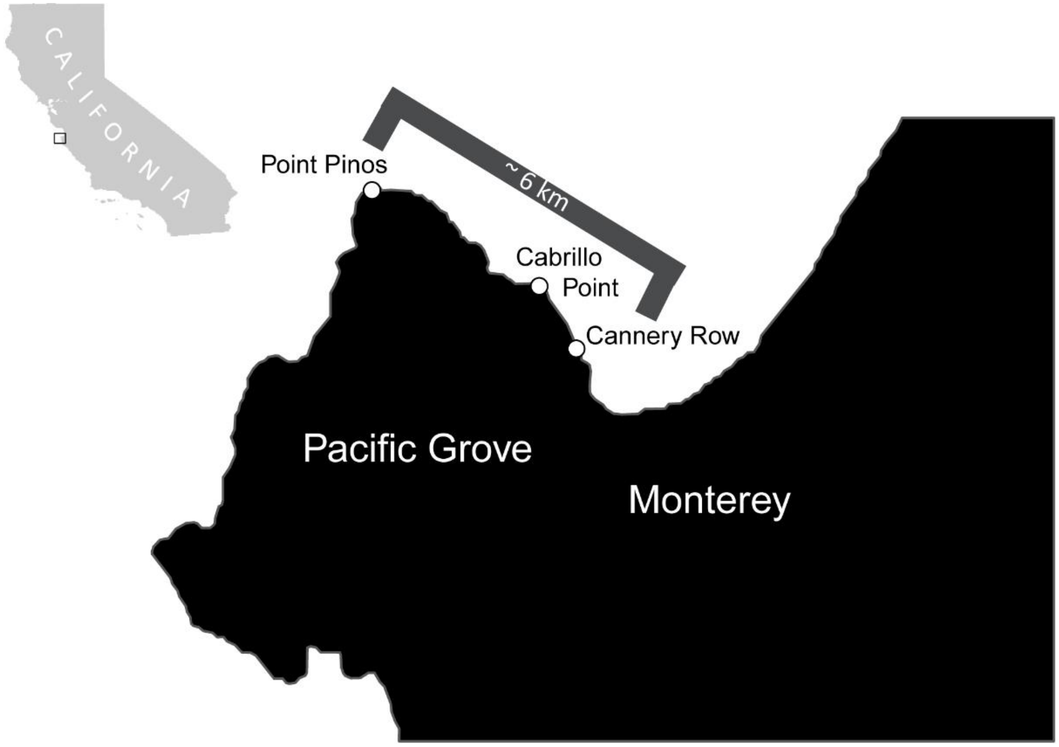

Figure S1. Map of study region. We collected macroalgae from Cabrillo Point (36.62, -121.90) for the curing experiment in May 2018. Multiple herbaria provided historical specimens that were collected from Point Pinos, Pacific Grove to Cannery Row, Monterey. Several early specimens were only identified to city but most collecting occurred in this region.

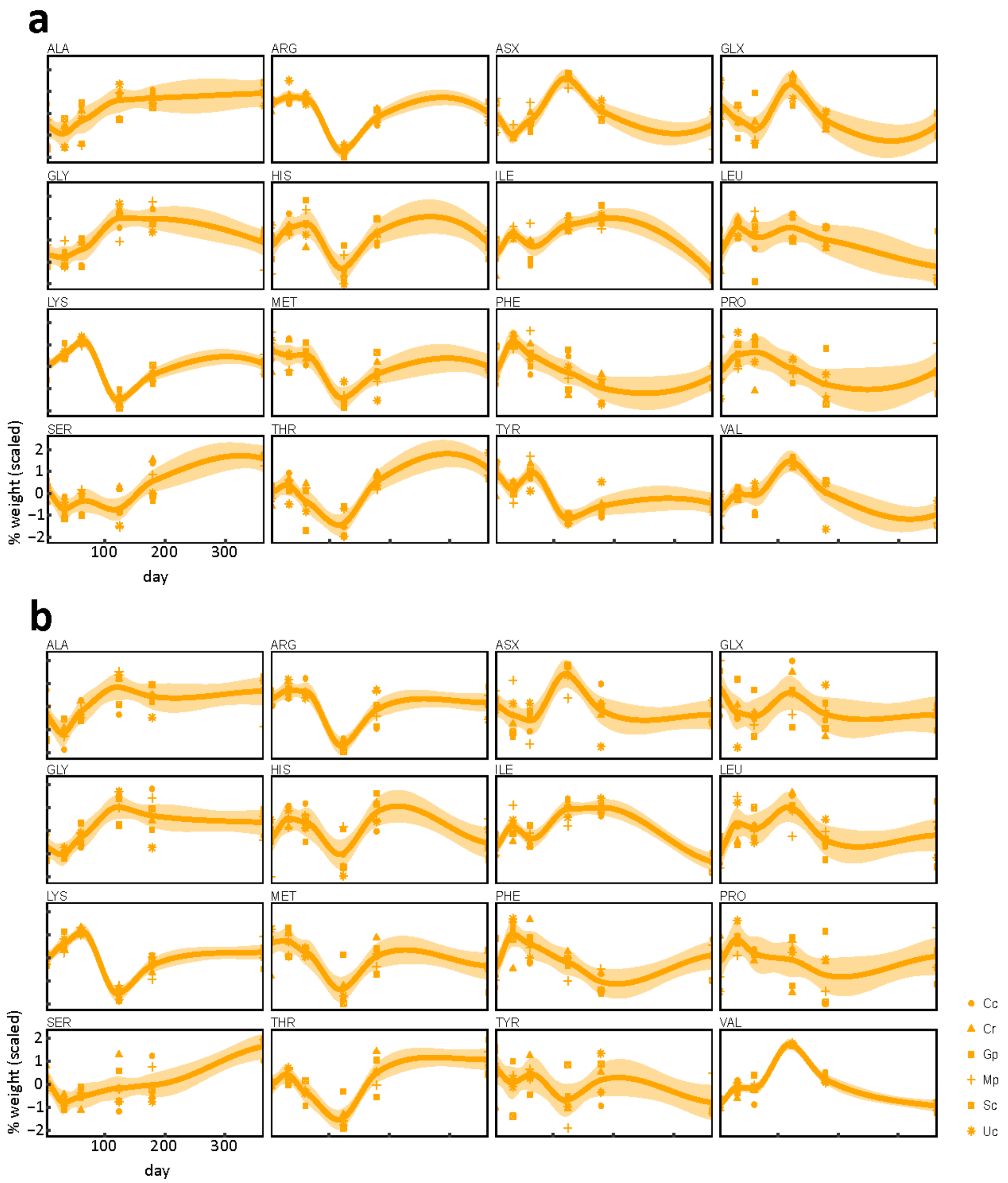

Figure S2. Amino acid composition of macroalgae on archived (a) and modern (b) paper over one year curing experiment. All species were combined for loess models and values were rescaled (see Methods).

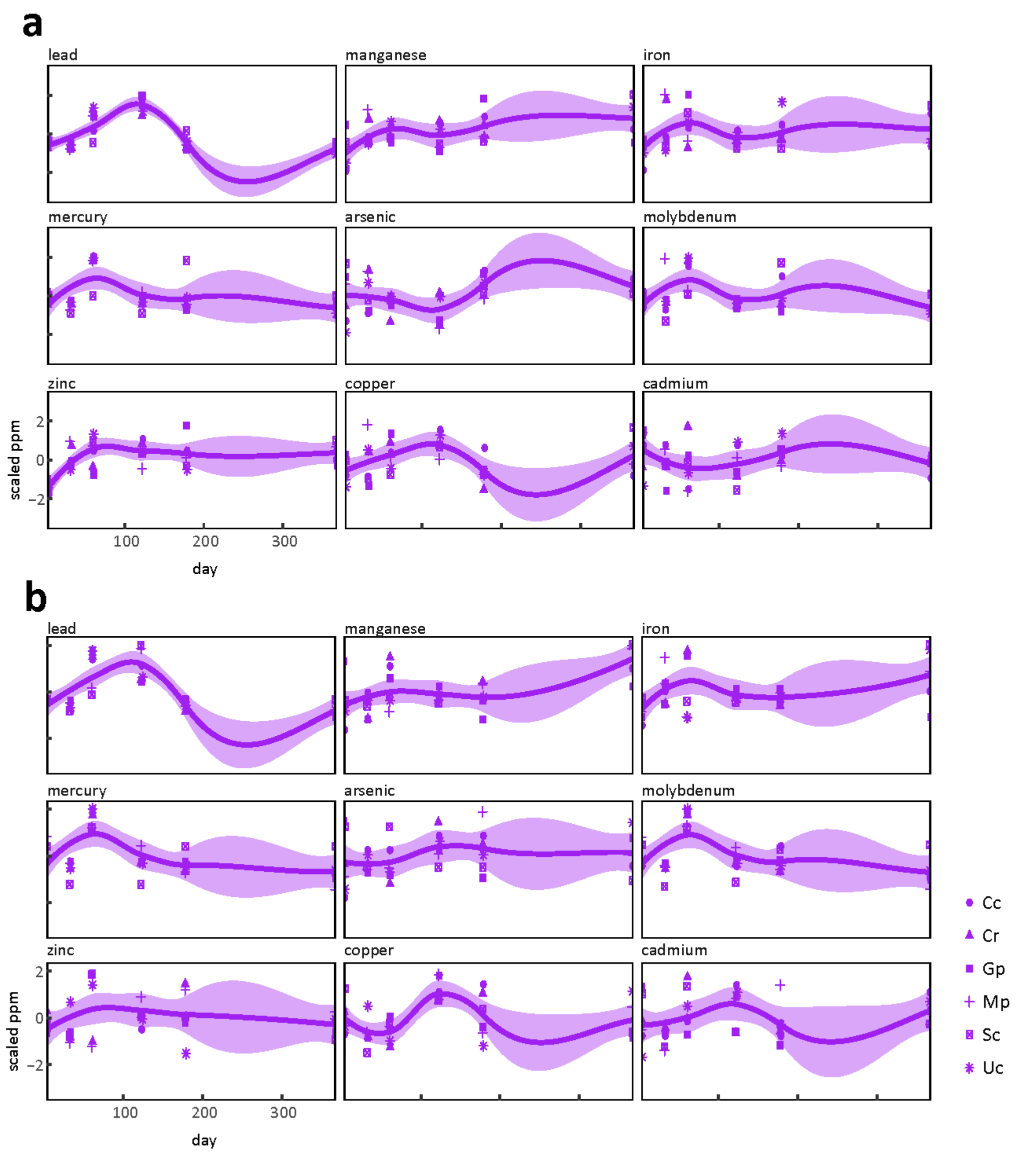

Figure S3. Heavy metal concentration of macroalgae on archived (a) and modern (b) paper over one year curing experiment. All species were combined for loess models and values were rescaled (see Methods).

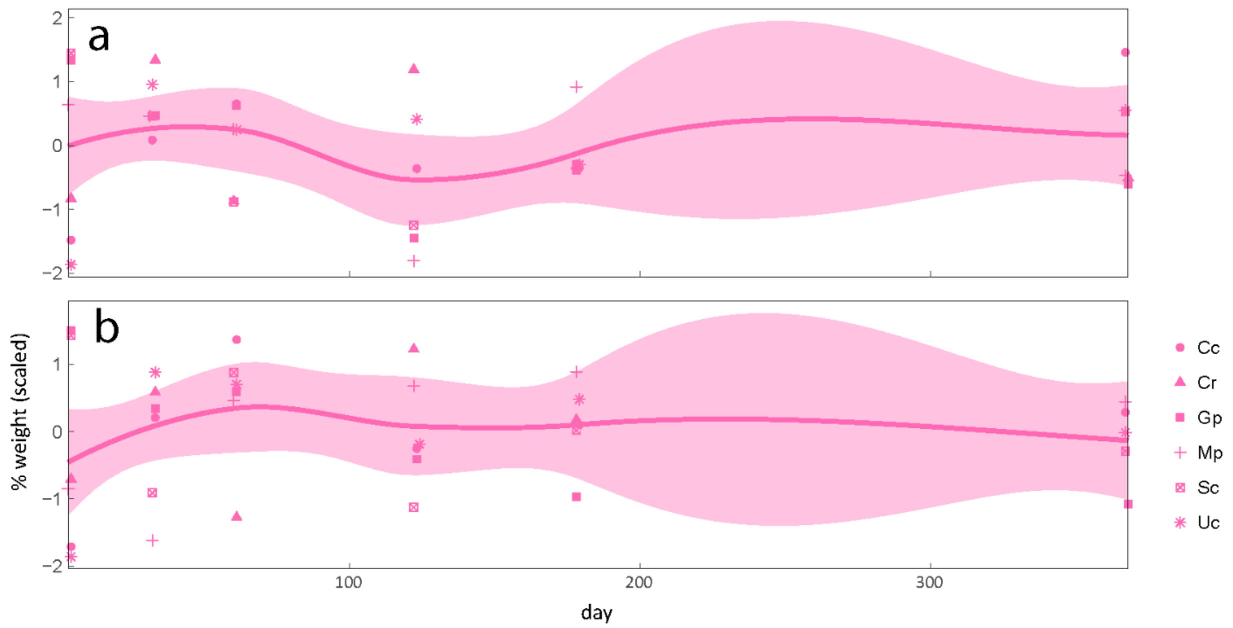

Figure S4. Total protein composition of macroalgae on archived (a) and modern (b) paper combined over one year curing experiment. All species were combined for loess models and values were rescaled (see Methods).

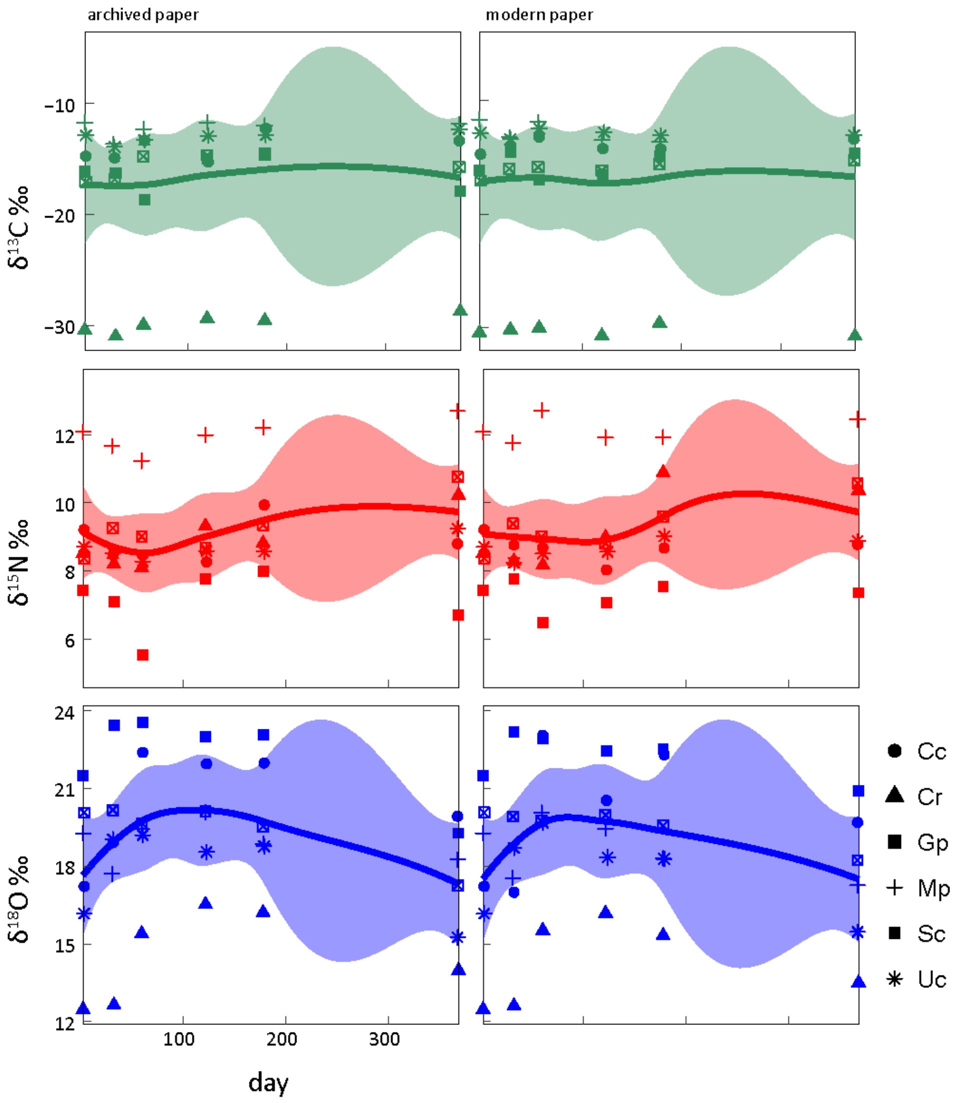

Figure S5. Stable isotope values of macroalgae on archived and modern paper over one year curing experiment. Loess curves are combined for all species.

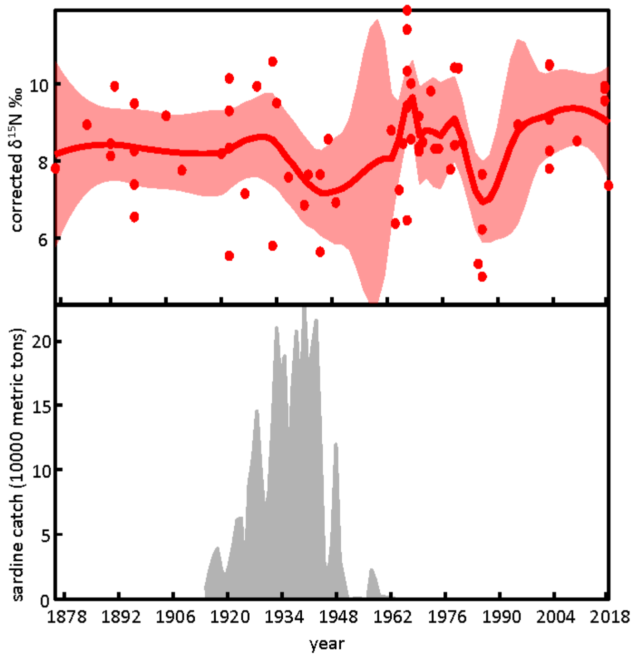

Figure S6. $\delta^{15}N$ values of *G. purpurascens* collected in southern Monterey Bay and recorded commercial sardine catch in Monterey were not correlated over the 140 year period. A loess model was fit to $\delta^{15}N$ values to visualize local trends.

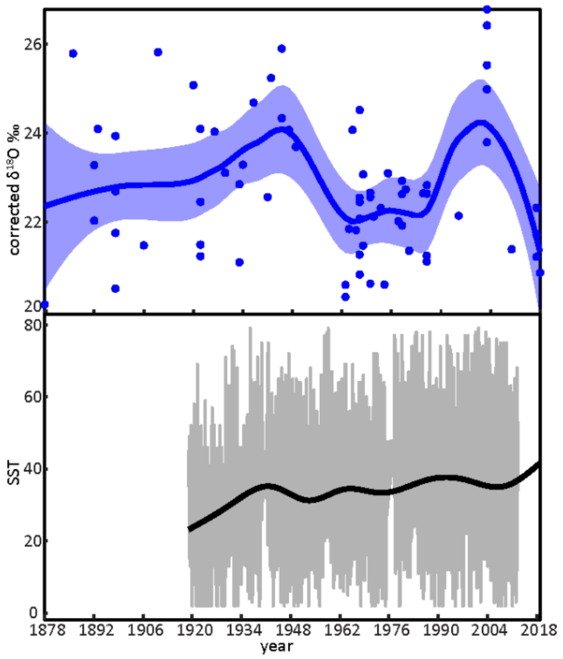

Figure S7. $\delta^{18}O$ values of *G. purpurascens* collected in southern Monterey Bay and sea surface temperature collected at Hopkins Marine Station were not correlated over the 140 year period. Loess models were fit to each data set to visualize local trends.

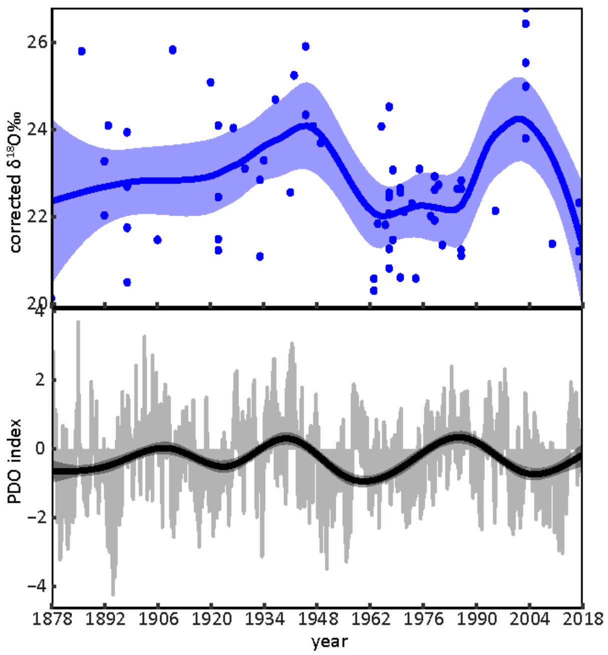

Figure S8. $\delta^{18}O$ values of *G. purpurascens* collected in southern Monterey Bay and calculated PDO index values for the region were not correlated over the 140 year period. Loess models were fit to each data set to visualize local trends.

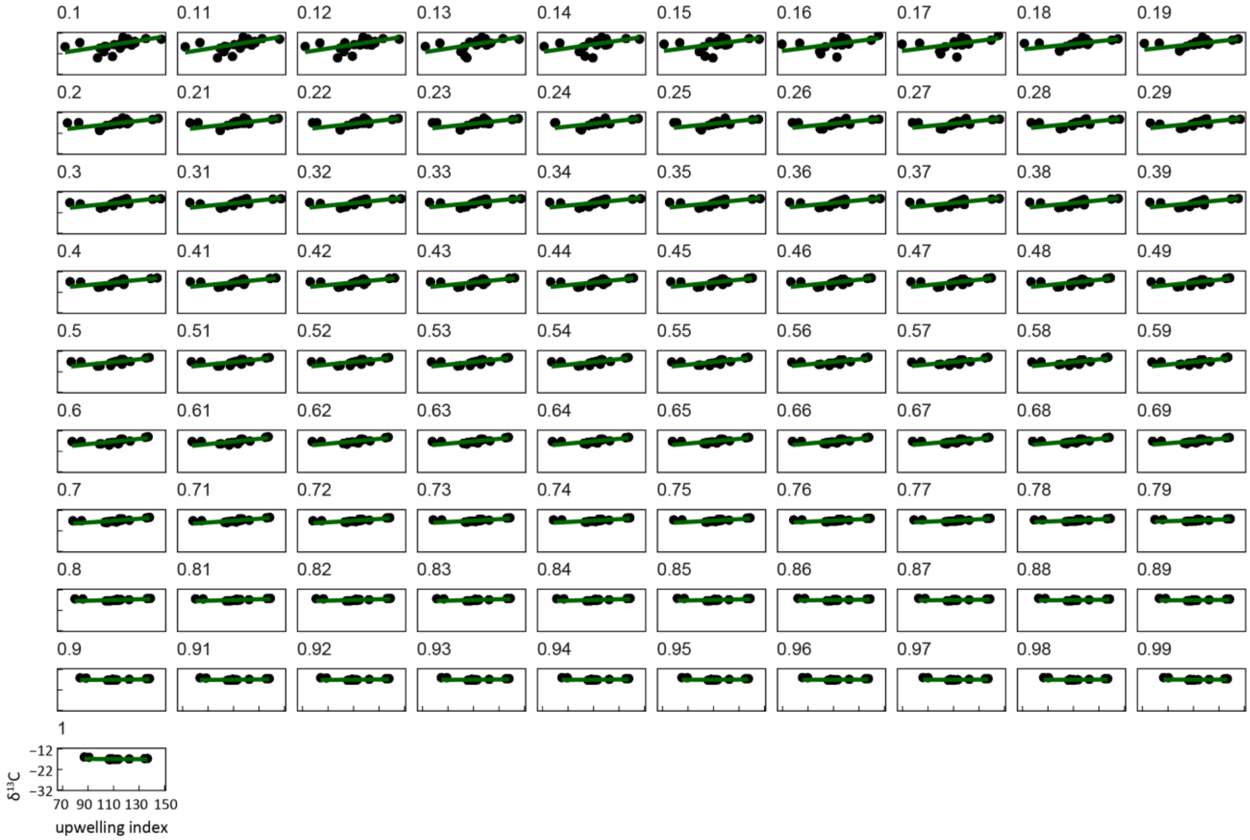

Figure S9. Modeled fits of stable carbon isotope ratios ($\delta^{13}C$) against modeled upwelling index values with varying spans. We did not have a priori expectations of biologically- or oceanographically-appropriate loess spans and therefore fit loess models using spans ranging from 0.1 to 1 by an interval of 0.01. We plotted the upwelling index loess fits against each stable isotope loess fit to examine the linear regressions for each span.

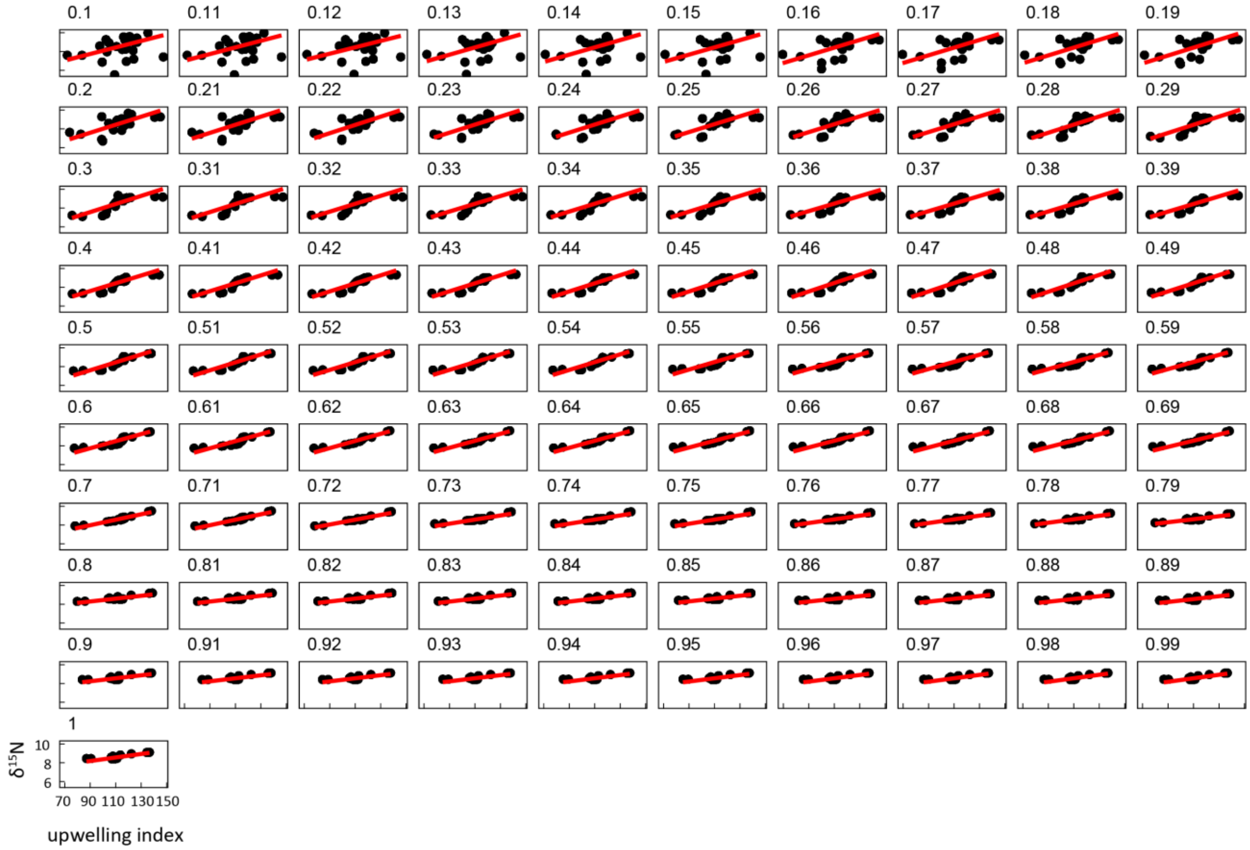

Figure S10. Modeled fits of stable nitrogen isotope ratios ($\delta^{15}N$) against modeled upwelling index values with varying spans. We did not have a priori expectations of biologically- or oceanographically-appropriate loess spans and therefore fit loess models using spans ranging from 0.1 to 1 by an interval of 0.01. We plotted the upwelling index loess fits against each stable isotope loess fit to examine the linear regressions for each span.

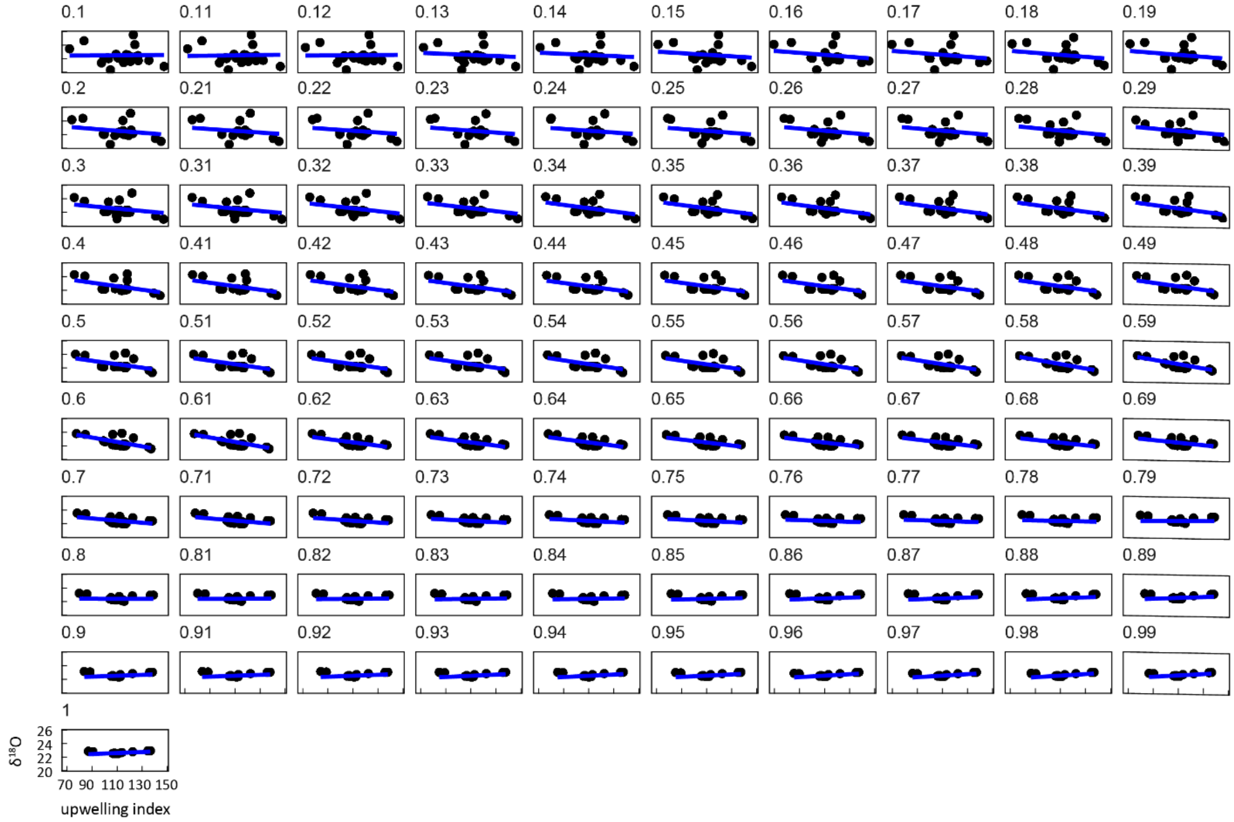

Figure S11. Modeled fits of stable oxygen isotope ratios ($\delta^{18}O$) against modeled upwelling index values with varying spans. We did not have a priori expectations of biologically- or oceanographically-appropriate loess spans and therefore fit loess models using spans ranging from 0.1 to 1 by an interval of 0.01. We plotted the upwelling index loess fits against each stable isotope loess fit to examine the linear regressions for each span.

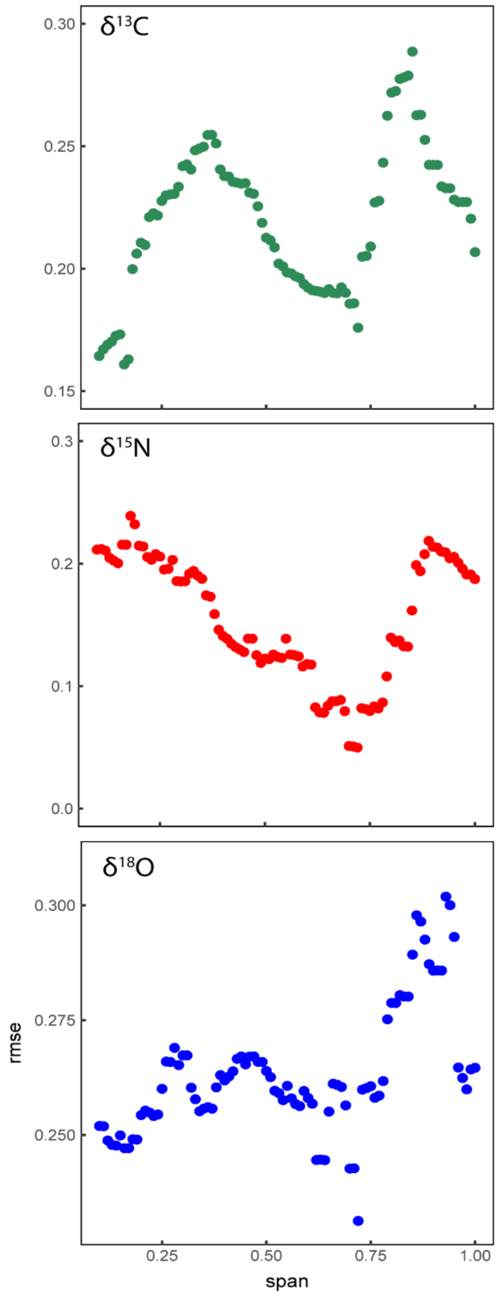

Figure S12. Optimal spans for loess models of historical macroalgae specimens and loess models of upwelling index values. The span with the lowest root mean square error (rmse) was determined to be the model that best fit the data.

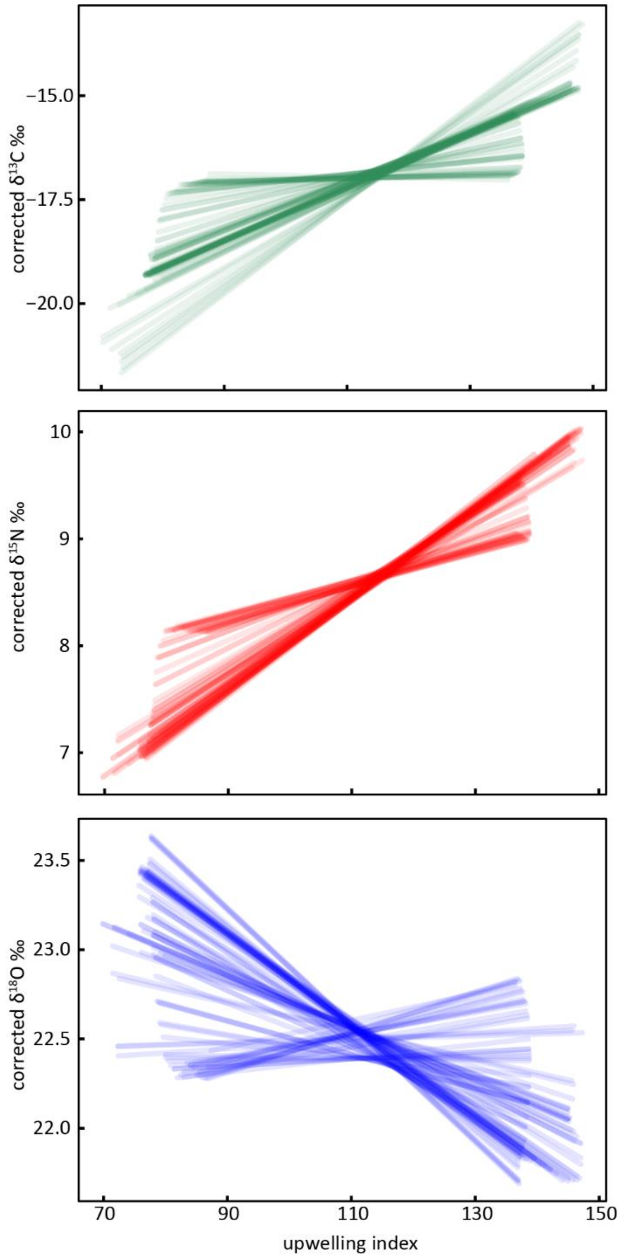

Figure S13. Relationship of stable isotope ratios from herbarium specimens to Bakun upwelling index. Loess model fits of macroalgae stable isotope values were plotted against loess model fits of upwelling index values. This was repeated with loess span values of 0.1-1 at an interval of 0.01. Linear regressions were fit to each span value and are shown above for $\delta^{13}C$, $\delta^{15}N$, and $\delta^{18}O$.
